# A cross-sectional study on assessment of spiritual health and its associated factors among undergraduate students in a medical college in Mangalore, Karnataka

**DOI:** 10.12688/f1000research.128273.1

**Published:** 2022-12-21

**Authors:** Pracheth Raghuveer, Ravjot Bhatia, Rohith Motappa, Malavika Sachith

**Affiliations:** 1 Epidemiology, National Institute of Mental Health and Neurosciences, India, Bangalore, Karnataka, 560029, India; 2 Community Medicine, Kasturba Medical College Hospital, Mangalore, Karnataka, 575001, India; 3 Community Medicine, P K DAS Institute of Medical Sciences, Ottapalam, Kerala, 679522, India

**Keywords:** Spirituality, Medical Students, Dakshina Kannada

## Abstract

**Background:** Spirituality and spiritual health are an integral component of an individual’s health and wellbeing. Among medical undergraduates and professionals, spiritual health has positive effects on the individual by decreasing burnout, psychological distress, and rates of substance abuse. Spiritual health is also correlated with increased satisfaction and meaning in life. Spiritual health also influences future patient care, builds patient- provider communication channels, and improves patient outcomes.

**Methods:** A cross-sectional study was conducted to assess the spiritual health levels of medical undergraduates in a medical college, in Karnataka, India, and to identify the factors associated with it. Using a pre-designed, validated semi-structed questionnaire, 600 medical undergraduates were approached and provided the forms. Spiritual health was assessed across the three domains of self-development, self-actualization, and self-realization using the Spiritual Health Assessment Scale (SHAS).

**Results:** A total of 436 medical undergraduates participated. Majority (67.7%) of participants were girls. Most (62%) were found to have fair spiritual health with a large portion of the rest (36%) having good spiritual health and 2% had poor spiritual health. A significant association was found  between spiritual health and the father’s (p=0.005) and mother’s (p=0.012) education levels. Spiritual health was also found to be associated with living in a nuclear family (p=0.04).

**Conclusions:** Majority of the medical undergraduates had fair spiritual health. Parents' education levels and nuclear family were significantly associated with spiritual health.

## Introduction

Spirituality, in its purest form, has been a vital aspect of an individual’s health since time immemorial. Health, often understood as a singular entity, is made up of multiple dimensions, with the WHO defining it as “a state of being where an individual is able to deal with day-to-day life in a manner which leads to the realization of one’s full potential; meaning and purpose of life; and happiness from within.” ^1^ While the three classical dimensions of health are rather extensive, there exists a fourth dimension, namely spiritual health. ^2^^,^^3^ Spiritual health, as defined by Dhar *et al.*, is “a state of being where an individual is able to deal with day-to-day life in a manner which leads to the realization of one’s full potential; meaning and purpose of life; and happiness from within.” Under this broad canopy of spiritual health, three aspects have been suggested which are, “Realization of one’s Full Potential”; “Meaning and Purpose of Life”; and “Happiness From Within”. ^3^

Over the last few decades, researchers have explored the relationships that exist between spiritual health Deb *et al.* c identified a significant positive correlation with finding satisfaction and meaning in life. ^4^ Spirituality has also been associated with a decreased risk for burnout among physicians and trainees. ^5^ A similar correlation has been observed among medical students, with higher levels of spirituality being associated with lower levels of burnout and psychological distress, with spirituality possibly acting as a protective factor. ^6^ Few studies have also found an inverse correlation between substance abuse and spirituality in college and medical students, with higher levels of spirituality correlating with lower rates of substance abuse as well as rates of binge drinking. ^7^^,^^8^

Beyond the overall personal wellness of the students, spiritual health also affects physicians and the care and treatment provided by them. In an article by Isaac *et al.*, spiritual health was found to be related to the interpretation of illnesses by patients. They also identified spirituality to have a mixed effect on health behaviour changes, acting both as a positive as well as a negative factor. Spirituality may also act as a bridge, furthering the patient – provider relationship and improving healthcare outcomes for all. ^9^ Geriatric care and palliative care may especially benefit from a physician’s spiritual health, by helping them explore and build a healthy connection with the patient, encouraging open and effective communication. Communication and openness play a vital role in patient centred care, and boost health outcomes. ^10^

The importance of spirituality for the wellness of a medical student as well as its impact on their care taking abilities cannot be understated. High level of spiritual health will improve the overall wellness of doctors, as well as the quality of care provided by them. While a few studies have been conducted to assess factors associated with spirituality, they are far and few in between, and even fewer exist for this region, and for this demographic that may be disproportionately benefited from spiritual wellness. Therefore, we carried out this study to assess the spiritual health of undergraduate medical students and the factors associated with it.

## Methods

The study was conducted on the undergraduate medical students of a medical college in south India. Ethical clearance was taken from the Institutional Ethical committee and all medical students of 4 batches (150×4) i.e. 600 students were approached for the study. Complete enumeration was done to include all medical students of Yenepoya Medical College from First to Third year of Bachelor of Medicine and Bachelor of Surgery (MBBS) course. Each batch has 150 students. So, the total sample size was 600. The study was carried out between December 2020 to January 2021. Those unwilling to give consent, those who did not own a phone or did not understand English were excluded from the study.

A pre-designed, validated, semi-structured proforma was prepared. This proforma was incorporated into a Google form. This was sent to the class representatives of each batch requesting them to distribute among their batchmates. The form had a participant information sheet and informed consent followed by the pre-designed questionnaire. The questionnaire included demographic variables like age, sex, religion, year of study. Spiritual Health Assessment Scale (SHAS) was be used to assess the three domains of spiritual health like self-development, self-actualization and self-realization. ^11^ It involves 21 questions, individual scores in SHAS ranges from 21 to 105 and the level of spiritual health can be further graded as poor (21 to 49), fair (50 to 77) and good (78 to 105). Questions pertaining to the factors associated with it were also included.

The Google form responses were downloaded in a comma separated value format and the data captured was further cleaned. Descriptive statistics like mean, frequency and proportion were applied. Chi-square test were used to assess the association between level of spiritual health and factors associated with it.

Clearance from the Institutional Ethics Committee was obtained. Permission from the Principal of the medical college to carry out the study was obtained. Written informed consent was obtained from all the study participants. Strict confidentiality of the information collected was maintained. All the data was kept confidential.

## Results

In the study, a total of 600 students were approached for the study, out of which 436 students responded. The response rate was 72.7%. The sociodemographic data is shown in Table 1. Most of the students (69.7%), belonged to female gender. About half the students (49.1%) students were Muslims while Hindus constituted 41.3% of the participants. Participation from the final year students was the least at 19%. A large majority (85.8%) were in a nuclear family while 14.2% were part of a joint family.

**Table 1.** Sociodemographic features of the medical students (N=436).

	N	%
**Gender**		
Male	132	30.3
Female	304	69.7
**Religion**		
Hindu	180	41.3
Christian	38	8.7
Muslim	214	49.1
Others	4	0.9
**Year of study**		
First year	107	24.5
Second year	138	31.7
Third year	108	24.8
Final year	83	19
**Type of family**		
Nuclear family	374	85.8
Joint family	62	14.2
**Father’s education**		
Uneducated	3	0.7
Schooling	78	17.9
Graduate	203	46.6
Post graduate and above	152	34.9
**Mother’s education**		
Uneducated	5	1.1
Schooling	116	26.6
Graduate	207	47.5
Post graduate and above	108	24.8
**Father’s occupation**		
Government	87	20
Private	154	35.3
Self employed	162	37.2
Retired	31	7.1
Unemployed	2	0.5
**Mother’s occupation**		
Government	72	16.5
Private	60	13.8
Self employed	67	15.4
Retired	8	1.8
Unemployed	229	52.5

Fathers’ education was analysed, and it was found that 46.6% were graduates, 34.9% were postgraduates and above. On the other hand, 47.5% mothers were graduates while 24.8% were postgraduates. Majority of the fathers (37.2%) were self-employed. More than half (52.5%) of the mothers were homemakers.

Table 2 shows the range of spiritual health obtained by the students. Self-development varied from 11 to 34 with a median of 26. Range of self-actualisation score was 7 to 35 and median was 26. Final spiritual health score varied from 40 to 103 with a median of 74.

**Table 2.** The range of spiritual health scores obtained by the medical students.

Spiritual health variables	Median (IQR)	Range of scores found in the survey	
		Maximum value	Minimum value
Self-development	26 (4)	34	11
Self-actualisation	26 (5)	35	7
Self-realisation	23 (6)	35	7
Final spiritual health score	74 (14)	103	40

**Figure 1.**
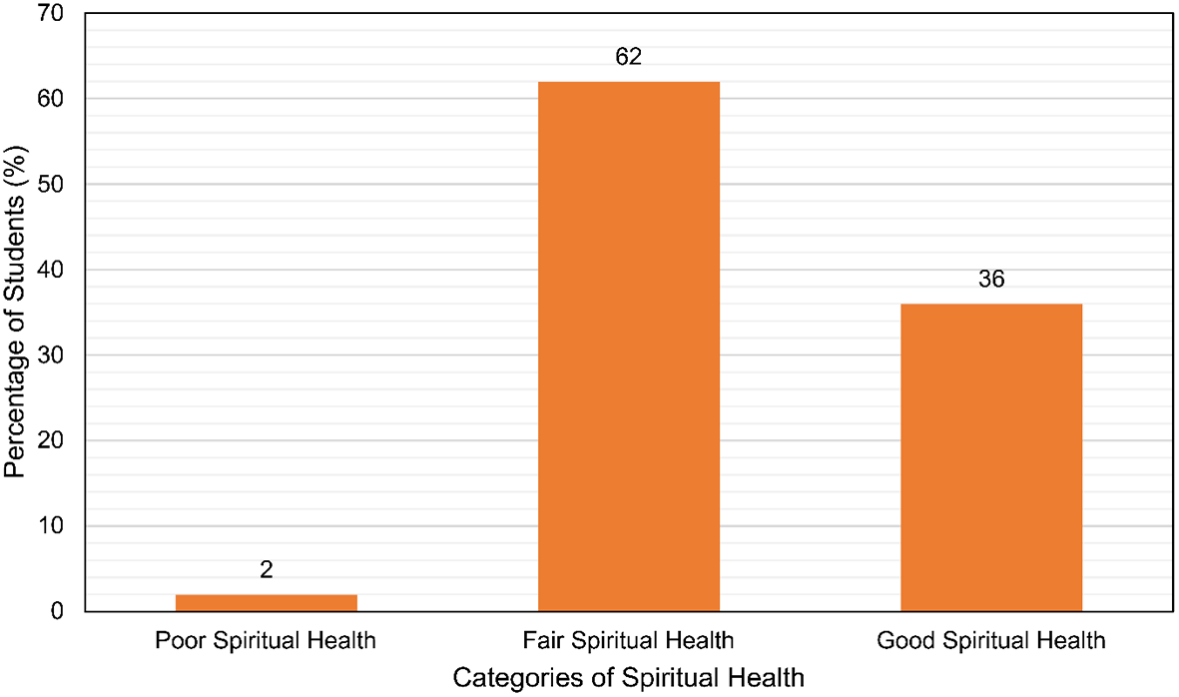
The spiritual health levels of the undergraduate medical students.

 It was found that a graphical representation of the findings is displayed in Figure 1.

Using the chi-square test, association of various demographic factors with spiritual health was analysed and is shown in Table 3. Upon analysis, out of all the variables studied, three of them were found to display significant association. Type of family(p<0.05), father’s education level and mother’s education level were found to be significantly associated with spiritual health. On the other hand, the other studied variables i.e., gender, religion, year of study, occupation of father and mother were not associated with spiritual health.

**Table 3.** Association of various demographic factors and spiritual health (N=436).

S.No	Variable		Categories of spiritual health			p- value *
			Poor	Fair	Good	
1.	Gender	Male (n=132)	4 (3.03%)	74 (56.06%)	54 (40.90%)	0.2
		Female (n=304)	4 (1.31%)	195 (64.14%)	105 (34.53%)	
2.	Religion	Hindu (n=180)	3 (1.66%)	123 (68.33%)	54 (30.0%)	0.1
		Christian (n=38)	2 (5.26%)	24 (63.15%)	12 (31.57%)	
		Muslim (n=214)	3 (1.40%)	119 (55.60%)	92 (42.99%)	
		Others (n=4)	0 (0)	3 (75.0%)	1 (25.0%)	
3.	Year of study	First year (n=107)	1 (0.93%)	70 (65.42%)	36 (33.64%)	0.56
		Second year (n=138)	2 (1.44%)	83 (60.14%)	53 (38.40%)	
		Third year (n=108)	3 (2.77%)	71 (65.74%)	34 (31.48%)	
		Final year (n=83)	2 (2.40%)	45 (54.21%)	36(43.37%)	
4.	Type of family	Nuclear family (n=374)	8 (2.13%)	237 (63.36%)	129 (34.49%)	**0.04** ^**#**^
		Joint family (n=62)	0 (0)	32 (51.61%)	30 (48.38%)	
5.	Father’s education	Uneducated (n=3)	1 (33.33%)	1 (33.33%)	1 (33.33%)	**0.005** ^**#**^
		Schooling (n=78)	1 (1.28%)	46 (58.97%)	31 (39.74%)	
		Graduate (n=203)	2 (0.98%)	128 (63.05%)	73 (35.96%)	
		Postgraduate and above (n=152)	4 (2.63%)	94 (61.84%)	54 (35.52%)	
6.	Mother’s education	Uneducated (n=5)	1 (20.0%)	2 (40.0%)	2 (40.0%)	**0.012** ^**#**^
		Schooling (n=116)	0 (0)	64 (55.17%)	52 (44.82%)	
		Graduate (n=207)	2 (0.96%)	137 (66.18%)	68 (32.85%)	
		Postgraduate and above (n=108)	5 (4.62%)	66 (61.11%)	37 (34.25%)	
7.	Father’s occupation	Government (n=87)	2 (2.29%)	56 (64.36%)	29 (33.33%)	0.98
		Private (n=154)	2 (1.29%)	96 (62.33%)	56 (36.36%)	
		Self employed (n=162)	3 (1.85%)	96 (59.25%)	63 (38.88%)	
		Retired (n=31)	1 (3.22%)	20 (64.51%)	10 (32.25%)	
		Unemployed (n=2)	0 (0)	1 (50.0%)	1 (50.0%)	
8.	Mother’s occupation	Government (n=72)	2 (2.77%)	50 (69.44%)	20 (27.77%)	0.50
		Private (n=60)	2 (3.33%)	39 (65.0%)	19 (31.66%)	
		Self employed (n=67)	2 (2.98%)	38 (56.71%)	27 (40.29%)	
		Retired (n=8)	0 (0)	4 (50.0%)	4 (50.0%)	
		Unemployed (n=229)	2 (0.87%)	138 (60.26%)	89 (38.86%)	

* Chi-square test.

^#^ Statistically significant.

## Discussion

We studied a variety of demographic factors to identify the ones which share any correlation with spiritual health. A majority of the students were female. Most of the respondents were believers of Hinduism or Islam, with Christians filling in most of the remaining percentage. A roughly equal breakdown of responses were seen across the different years of study. Most of the respondents live in a nuclear family.

Out of all the variables assessed, fathers’ and mother’s education levels were found to be significantly associated with spiritual health. This result is similar to a study conducted by Kalpana *et al.*, among arts and science college students. ^12^ This could indicate and uncover the role a person’s family background plays in the spiritual wellness of an individual. The adage “education begins at home”, could very well be applied here. Parents and families are the first teachers of children. The foundational knowledge of a person is to a large extent influenced by the teachings of their parents and their family environment. Among other things, spiritual wellness and its associated techniques are often taught at home, with parents acting as the conduits of spirituality. Old beliefs, customs and rituals are passed down from generation to generation and play a vital role in the healthy exploration of spirituality.

Education and it's association with spiritual health should not be ignored. Teaching spiritual health components and techniques could be a possible way to counter and improve upon existing deficits in this aspect. The role of education should be considered and requires further exploration.

A significant association was noted between spirituality and nuclear families. This is unlike the findings of one study conducted by Ahangar *et al.*, which found a higher level of spirituality in adolescents living in joint families, compared to nuclear families. ^13^ This could possibly be due to the fact that the college is located in an urban area, which may have more nuclear families. Another factor is that due to the type of college, most of the students belong to higher socioeconomic stratas, which are often associated with independent nuclear family lifestyles.

In our study, 36% of the students had good spiritual health while 62% had fair spiritual health. 2% of the students had poor spiritual health. This proportion is somewhat similar to another study, which found a similar breakdown among dental college students, with the majority (74.55%) having fair spiritual health. ^14^ This could be attributed to a similar demographic of the respondents, and supports and strengthens the outcomes of this study.

A few studies have found gender to be a significant variable which we could not identify with significance. ^14^^–^^16^ The rest of the variables were not found to have any significant association.

The lack of a significant association between religion and spirituality could hint towards the general nature of spiritual health regardless of the conforming religion of the respondents. Common approaches could thus possibly be designed and implemented in order to boost spiritual health among the surveyed demographic.

Overall, few studies have been conducted in order to assess the variables associated with this oft neglected domain of spiritual health and wellness. As mentioned previously, the effects of spiritual health reach far and beyond, especially for medical students and future physicians. Further research would be required in order to solidify the associations as well as identify other possible factors influencing and affecting spiritual health.

### Strengths and limitations

Our study had several strengths. The large sample size afforded allows us a greater degree of reliability and the ability to generalize the results to other similar demographics. The design of this study also makes it easy to replicate and conduct further assessments across other demographics, as well as solidify the associations.

The limitations of this study include the fact that spiritual health has different definitions for different respondents, and thus the interpretation of the questions could vary from respondent to respondent, based on their own experiences with it. The second limitation is that this is a cross sectional study, and thus provides possible correlations and associations only. Another limitation that arises is that Yenepoya Medical College, a prestigious medical college in Mangalore, India, is a private college. Private colleges usually have higher fee structures associated with them; thus, the students usually belong to a higher socioeconomic status than government colleges, which have a healthy mix. Therefore, the results, which while useful and suitable for this demographic, cannot be fully generalized to students in all kinds of colleges. Nevertheless, spiritual health, the focus of our study, could play an important role in the overall health of a medical student as well as the outcomes of patient-centred cared that may be provided by the student in future.

## Conclusion

 Majority of the undergraduate medical students were found to have spiritual health. We have analyzed a few variables that could have possibly been associated with spiritual health. It was found that parents’ education levels as well as the family type have been identified as possible factors affecting spiritual well-being. These factors can be targeted in order to improve the spiritual health. Early identification of these variables can allow us to develop newer programs and approaches which can be used to improve the students’ spiritual health.

## Data Availability

Figshare: A cross sectional study on assessment of spiritual health and its associated factors among undergraduate students in a medical college in Mangalore, Karnataka (Responses) (2).xlsx, https://doi.org/10.6084/m9.figshare.21509781.v1. ^17^ Figshare: A cross-sectional study on assessment of spiritual health and its associated factors among undergraduate students in a medical college in Mangalore, Karnataka. Informed Consent form.docx, https://doi.org/10.6084/m9.figshare.21601029.v1. ^18^ Figshare: A cross-sectional study on assessment of spiritual health and its associated factors among undergraduate students in a medical college in Mangalore, Karnataka. Participant Information Sheet.docx, https://doi.org/10.6084/m9.figshare.21601023. ^19^ Figshare: A cross-sectional study on assessment of spiritual health and its associated factors among undergraduate students in a medical college in Mangalore, Karnataka. Questionnaire.docx, https://doi.org/10.6084/m9.figshare.21601005.v1. ^20^ Data are available under the terms of the Creative Commons Attribution 4.0 International license (CC-BY 4.0).
