# Supplementary material for: A cross-sectional study on assessment of spiritual health and its associated factors among undergraduate students in a medical college in Mangalore, Karnataka
Source: F1000Res. 2023 Jul 4;11:1546. Originally published 2022 Dec 21. [Version 2] doi: 10.12688/f1000research.128273.2 (PMC10352630; doi:10.12688/f1000research.128273.2)
Supplement: Peer review history

# Peer review: Reviewer response for version 2

**DOI:** 10.5256/f1000research.148603.r183926

**Published:** 2023-07-17

**Authors:** Vedalaveni Chowdappa Suresh

**Affiliations:** 1 Department of Psychiatry, Akash Institute of Medical Sciences and Research Centre, Bangalore, Karnataka, India

No further comments to make.

Is the work clearly and accurately presented and does it cite the current literature?

Yes

If applicable, is the statistical analysis and its interpretation appropriate?

Yes

Are all the source data underlying the results available to ensure full reproducibility?

Yes

Is the study design appropriate and is the work technically sound?

Yes

Are the conclusions drawn adequately supported by the results?

Partly

Are sufficient details of methods and analysis provided to allow replication by others?

Yes

Reviewer Expertise:

Mental Health

I confirm that I have read this submission and believe that I have an appropriate level of expertise to confirm that it is of an acceptable scientific standard.

# Peer review: Reviewer response for version 2

**DOI:** 10.5256/f1000research.148603.r183925

**Published:** 2023-07-11

**Authors:** Chonnakarn Jatchavala

**Affiliations:** 1 Department of Psychiatry, Prince of Songkla University, Songkhla, Thailand

I am very satisfied with the revision. Nothing is to be changed or further revised.

Is the work clearly and accurately presented and does it cite the current literature?

Yes

If applicable, is the statistical analysis and its interpretation appropriate?

Partly

Are all the source data underlying the results available to ensure full reproducibility?

Partly

Is the study design appropriate and is the work technically sound?

Yes

Are the conclusions drawn adequately supported by the results?

Partly

Are sufficient details of methods and analysis provided to allow replication by others?

Partly

Reviewer Expertise:

NA

I confirm that I have read this submission and believe that I have an appropriate level of expertise to confirm that it is of an acceptable scientific standard.

# Peer review: Reviewer response for version 1

**DOI:** 10.5256/f1000research.140845.r158713

**Published:** 2023-02-13

**Authors:** Chonnakarn Jatchavala

**Affiliations:** 1 Department of Psychiatry, Prince of Songkla University, Songkhla, Thailand

Major points: 

1. This study is relatively new, we are talking about spiritual health which is very controversial. And it is a broad range to discuss.
2. In the results section of the abstract, please add more findings relevant to the study objectives.
3. Discussing empirical implications in medical education, authors need to find other cultures' perspectives on spiritual health that decrease burnout/ mental health issues or increase positive factors in higher education.
4. Could you modify the conclusion and make it specific to the study objectives?
5. What are the recommendations based on the study findings? Please include them in the discussion.

Minor points:

1. Please give us the odds ratio for the significantly associated factor if possible 
2. Table 3 is quite enough. Table 2 could be noted: under table 2

Is the work clearly and accurately presented and does it cite the current literature?

Yes

If applicable, is the statistical analysis and its interpretation appropriate?

Partly

Are all the source data underlying the results available to ensure full reproducibility?

Partly

Is the study design appropriate and is the work technically sound?

Yes

Are the conclusions drawn adequately supported by the results?

Partly

Are sufficient details of methods and analysis provided to allow replication by others?

Partly

Reviewer Expertise:

Mental health, higher education

I confirm that I have read this submission and believe that I have an appropriate level of expertise to confirm that it is of an acceptable scientific standard, however I have significant reservations, as outlined above.

# Response

**Published:** 2023-05-22

**Authors:** Rohith Motappa

**Affiliations:** Kasturba Medical College Hospital, India

What are the recommendations based on the study findings? Please include them in the discussion  

 Response. Thank you for the comment.  Recommendations have been included in the discussion section last paragraph, Page 11.

 Rest all comments have been addressed in the modified article file

# Peer review: Reviewer response for version 1

**DOI:** 10.5256/f1000research.140845.r158712

**Published:** 2023-01-20

**Authors:** Vedalaveni Chowdappa Suresh

**Affiliations:** 1 Department of Psychiatry, Akash Institute of Medical Sciences and Research Centre, Bangalore, Karnataka, India

**Abstract:**

 Conclusion: can be improvised. As both results and conclusion depicts same content.

**Keywords:** can consider removing Dakshina Kannada.

**Introduction**

1. Definition of health [reference 1] need to be re-written.
2. Definition of Spiritual health defined twice [ two different references]
3. 2nd paragraph: relationship between spiritual health and what?

**Methodology:**

1. Any specific reason to include only 1st-3rd year students?
2. Ethical committee clearance mentioned twice. Since it is from same institution, need not mention about permission from principal in methods.
3. Study proforma had participant information sheet with **consent form **as mentioned, any reason for obtaining written consent separately?
4. Why was the domains like socio-economic status, background [rural/semiurban/urban] was not considered.

**Results:**

 Could mention the variables ie. gender, religion, year of study, occupation did not show any significant correlation with the spirituality.

Is the work clearly and accurately presented and does it cite the current literature?

Yes

If applicable, is the statistical analysis and its interpretation appropriate?

Yes

Are all the source data underlying the results available to ensure full reproducibility?

Yes

Is the study design appropriate and is the work technically sound?

Yes

Are the conclusions drawn adequately supported by the results?

Partly

Are sufficient details of methods and analysis provided to allow replication by others?

Yes

Reviewer Expertise:

Mental Health

I confirm that I have read this submission and believe that I have an appropriate level of expertise to confirm that it is of an acceptable scientific standard, however I have significant reservations, as outlined above.

# Response

**Published:** 2023-05-22

**Authors:** Rohith Motappa

**Affiliations:** Kasturba Medical College Hospital, India

The definition of health [reference 1] needs to be re-written

 Response:  The definition has been rewritten and  incorporated 

 2nd paragraph: the relationship between spiritual health and what?  

 Response: Thanks we have included it on page 2 of the manuscript in the introduction section, 3rd paragraph

 Ethical committee clearance mentioned twice. Since it is from same institution, need not  mention about permission from principal

 Response: Thanks we have made the changes as advised. Ethics Committee clearance is mentioned just once. Please find the changes in the methods section, first and third paragraphs. 

 Study proforma had participant information sheet with consent form as mentioned, any reason for obtaining written consent separately?

 Response: Thanks for the comment. We have removed the written consent part in methods section.
